# Double-Decker-Shaped Polyhedral Silsesquioxanes Reinforced Epoxy/Bismaleimide Hybrids Featuring High Thermal Stability

**DOI:** 10.3390/polym14122380

**Published:** 2022-06-12

**Authors:** Wei-Cheng Chen, Zih-Yu Chen, Yuxia Ba, Bingyang Wang, Guofei Chen, Xingzhong Fang, Shiao-Wei Kuo

**Affiliations:** 1 Department of Materials and Optoelectronic Science, College of Semiconductor and Advanced Technology Research, Center for Functional Polymers and Supramolecular Materials, National Sun Yat-Sen University, Kaohsiung 80424, Taiwan; d053100003@student.nsysu.edu.tw (W.-C.C.); b073100051@student.nsysu.edu.tw (Z.-Y.C.); 2 Dongying Xinbang Electronic Technology Co., Ltd., Dongying 257000, China; byx781217@163.com (Y.B.); wang8957wang@126.com (B.W.); 3 Ningbo Institute of Materials Technology and Engineering, Chinese Academy of Sciences, Ningbo 315201, China; gfchen@nimte.ac.cn; 4 Department of Medicinal and Applied Chemistry, Kaohsiung Medical University, Kaohsiung 807, Taiwan

**Keywords:** bismaleimide, epoxy, DDSQ, nanocomposites, thermal stability

## Abstract

In this study, we synthesized bismaleimide into a functionalized double-decker silsesquioxane (DDSQ) cage. This was achieved by hydrosilylation of DDSQ with nadic anhydride (ND), reacting it with excess *p*-phenylenediamine to obtain DDSQ-ND-NH_2_, and treating with maleic anhydride (MA), which finally created a DDSQ-BMI cage structure. We observed that the thermal decomposition temperature (*T*_d_) and char yield were both increased upon increasing the thermal polymerization temperature, and that these two values were both significantly higher than pure BMI without the DDSQ cage structure since the inorganic DDSQ nanoparticle could strongly enhance the thermal stability based on the nano-reinforcement effect. Based on FTIR, TGA, and DMA analyses, it was found that blending epoxy resin with the DDSQ-BMI cage to form epoxy/DDSQ-BMI hybrids could also enhance the thermal and mechanical properties of epoxy resin due to the organic/inorganic network formation created by the ring-opening polymerization of the epoxy group and the addition polymerization of the BMI group due to the combination of the inorganic DDSQ cage structure and hydrogen bonding effect. The epoxy/DDSQ-BMI = 1/1 hybrid system displayed high *T*_g_ value (188 °C), *T*_d_ value (397 °C), and char yield (40.4 wt%), which was much higher than that of the typical DGEBA type epoxy resin with various organic curing agents.

## 1. Introduction

Epoxy resin is one of the most important thermosetting resins and is especially useful for high-performance applications due to its good adhesion to most substrates, outstanding chemical resistance to solvents and moisture, and utility in various applications in composite, coating, painting, and insulating for semiconductor or electric devices [1,2,3,4,5,6]. Nonetheless, conventional epoxy resin cannot meet the requirements for thermal or flame resistance, and thus high thermal stability polymers such as poly(ether imide) or poly(ether sulfone) have been used in epoxy resin to enhance this property [7,8,9,10]. Furthermore, nanomaterials such as polyhedral oligomeric silsesquioxane (POSS), clay, or graphene have also been reported as viable modifications to the epoxy matrix because these inorganic materials usually possess a higher thermal stability than organic polymers [11,12,13,14,15,16].

Bismaleimide (BMI) is another widely used material in high-performance thermosetting resin in aerospace, electronic encapsulation, and printed-circuit board applications because of its high thermal stabilities, low flammability, and high electrical insulation [17,18,19,20]. Many commercially available bismaleimide-derivatives of resin are reported due to their simple industrial synthesis and cheap raw materials [21,22]. Many studies have investigated enhancing the physical properties of BMI in order to design reactive functional groups or rigid moieties into the BMI monomers, which could also incorporate inorganic nanoparticles such as POSS, graphene, or carbon nanotubes [23,24,25,26,27,28,29]. As mentioned above, conventional epoxy resins are limited by their low thermal stability, e.g., low glass transition temperature (*T*_g_), which means they cannot be safely be used at temperatures higher than 140 °C. On the contrary, BMI possesses high thermal stability; however, its poor processability as a solid compound is a disadvantage. As a result, the combination of both epoxy and BMI could help to make the most of the beneficial properties of these two thermosetting resins [30,31,32].

To further enhance the thermal properties of epoxy/BMI blends, the incorporation of inorganic nanoparticles such as POSS into the blend system is a reasonable approach that could also increase the oxidation resistance and decrease the surface free energy and flammability [33,34,35]. In general, polymer/POSS nanocomposites could be positioned at chain ends or sides by using the mono-functionalized POSS [36,37,38,39,40] or could act as the crosslinking agent to form network structures from the multi-functionalized POSS nanoparticles [41,42,43,44,45]. For example, octa-functionalized epoxy or maleimide based on POSS nanoparticles have been proposed [46,47,48], which could enhance its thermal stability into epoxy, phenolic, benzoxazine, and cyanate ester resins [49,50,51,52,53,54,55]. However, these octa-functionalized POSS compounds usually cannot crosslink well because of their 3D geometry structure, and these residue epoxy or maleimide functional units in thermosetting resin are not acceptable in real high-performance electronic applications [46,47,48]. Recently, double-decker–shaped polyhedral silsesquioxane (DDSQ) has been proposed as a bi-functionalized POSS nanoparticle added into polyimide, polyurethane, and polybenzoxazine resins to lower their dielectric constants by increasing the free volume [56,57,58,59,60,61,62,63]. In addition, DDSQ cage structures are highly thermally stable because of their intrinsic inorganic property, which improves the thermal stability of epoxy or BMI resins through chemical covalent bonds or physical dispersion [64,65].

**Figure 1:**
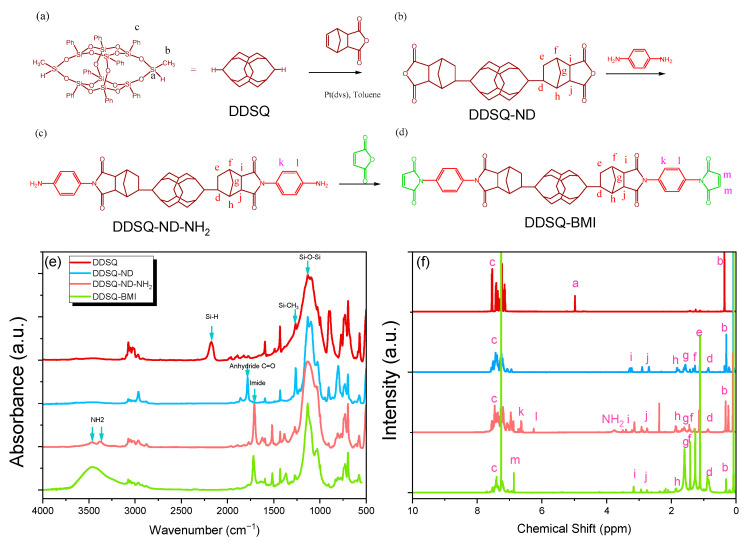
The synthesis of DDSQ-BMI (**d**) from (**a**) DDSQ, (**b**) DDSQ-ND, and (**c**) DDSQ-ND-NH_2_ monomer; the corresponding (**e**) FTIR and (**f**) ^1^H NMR spectra of each (**a**–**d**) compound.

Taking into account the chemical structure of typical bismaleimide of 1,4-bis(maleimido)benzene, its functional group is the benzene ring. To enhance its thermal property, the DDSQ cage structure was introduced into the BMI to replace the benzene ring and to form a new type of DDSQ-BMI hybrid. Therefore, incorporation of inorganic DDSQ nanoparticles may enhance the thermal properties of BMI resin and can then blend with epoxy resin. Thus, we firstly prepare a bi-functional amine DDSQ cage structure, as shown in Figure 1a–c in this study. The second step is to form a bismalemide DDSQ (DDSQ-BMI) through a reaction with malic anhydride (Figure 1d), which could be confirmed by FTIR and NMR spectroscopy analyses. Finally, the copolymerization of epoxy with BMI and DDSQ-BMI monomers successfully forms the network structure to achieve high thermal stability based on TGA and DMA analyses.

## 2. Experimental Section

### 2.1. Materials

Maleic anhydride (MA), *p*-phenylenediamine, toluene, acetone, N,N-diethylethanamine, and acetic anhydride were purchased from Sigma–Aldrich (Taipei, Taiwan). DDSQ-ND was synthesized as described previously (Figure 1b) [35,60]. The epoxy resin (DGEBA, DER 331) was purchased from Dow Chemical (Midland, MI, USA) where EEW is 190 g/eq [66].

### 2.2. Synthesis of DDSQ-ND-NH_2_

DDSQ-ND (2.96 g, 2 mmol) and the excess *p*-phenylenediamine (PPD, 1.30 g, 12 mmol) were placed under a blanket of N_2_. Toluene (60 mL) was added dropwise while stirring vigorously and heated to 105 °C for 48 h under N_2_ atmosphere. The solution was filtered, and the filtrates were concentrated by using vacuum distillation. It was then dried under vacuum oven at 180 °C to obtain a brown/red solid product; yield was 88%.

### 2.3. Synthesis of BMI and DDSQ-BMI

DDSD-ND-NH_2_ (3.324 g, 2 mmol) or *p*-phenylenediamine (PPD, 0.216 g, 2 mmol) and maleic anhydride (0.4116 g, 4.2 mmol) were placed into the flask with the reflux condenser. Next, 50 mL of dry acetone was added to the serum plug and stirred for about 30 min. At the same time, 40 mL acetone was taken to another beaker containing maleic anhydride. After dissolving, it was added to the flask through a titration funnel and stirred for 30 min. The mixture was heated to 40 °C and held for another 30 min. N,N-diethylethanamine 0.35 mL and acetic anhydride 0.15 g were added to the flask. After 24 h, unreacted solids were filtered out. A 1000 mL beaker with ice and deionized water was used to re-precipitate the crude product. The mixture was slowly dripped into the beaker, then stirred. After standing for 30 min, the precipitate was filtered and washed with water containing 10% sodium carbonate. It was then dried at room temperature in an oven for one day and provided BMI (Figure S1) or DDSQ-BMI compound; yield was 81%.

### 2.4. Preparation of Epoxy/BMI or Epoxy/DDSQ-BMI Hybrids

Various amounts of epoxy with BMI or DDSQ-BMI nanoparticle were stirred for 1 h at 60 °C and then degas under vacuum overnight. The cast samples were placed into the aluminum dish and thermal curing at 180, 240, and 300 °C each for 2 h.

## 3. Results and Discussion

### 3.1. Synthesis of DDSQ-Functionalized Bismaleimdie (DDSQ-BMI) Monomer

Figure 1a–d present the preparation of the DDSQ-BMI monomer. Each intermediate chemical structure could be confirmed by FTIR and ^1^H NMR analyses. Figure 1e shows the FTIR spectra of each DDSQ derivative obtained during the synthesis of DDSQ-BMI monomer; all DDSQ derivatives exhibit a weak signal at 1261 cm^−1^ due to the Si-CH_3_ unit, and a strong signal at 1097 cm^−1^ due to the Si-O-Si unit. After the hydrosilylation of DDSQ with ND, the Si-H absorption at 2172 cm^−1^ for pure DDSQ disappeared and then formed the anhydride C=O units at 1860 and 1782 cm^−1^ for DDSQ-ND, indicating complete hydrosilylation [60]. The FTIR spectrum of DDSQ-ND-NH_2_ shows the imide C=O units at 1708 and 1772 cm^−1^; the two absorptions at 3374 and 3460 cm^−1^ correspond to symmetric and asymmetric NH_2_ units, respectively. The C=O absorption reveals the lower wavenumber, and the NH_2_ units confirm the formation of DDSQ-ND-NH_2_ [60]. The slight red shift to 1718 and 1773 cm^−1^ of imide C=O units and the signals for NH_2_ units disappeared, suggesting the formation of DDSQ-BMI (Figure 1d).

Figure 1f also shows the corresponding ^1^H NMR spectra of each DDSQ derivative synthesized in this study. After the hydrosilylation of DDSQ with ND, the Si-H protons at 4.98 ppm for pure DDSQ disappeared and then formed aliphatic protons at 3.25–0.83 ppm with two isomers, also confirming the complete hydrosilylation of DDSQ-ND [60]. The DDSQ-ND-NH_2_ spectrum shows a broad peak at 3.74 ppm for the NH_2_ units, and two peaks at 6.25 and 6.62 ppm for the aromatic protons of *p*-phenylene diamine, indicating the formation of DDSQ-ND-NH_2_. The NH_2_ signal has disappeared, and the vinyl signals for the MA unit at 6.83 ppm for DDSQ-BMI also confirm the synthesis of DDSQ-BMI nanoparticles.

### 3.2. Thermal Curing Behavior of BMI and DDSQ-BMI Monomer

**Figure 2:**
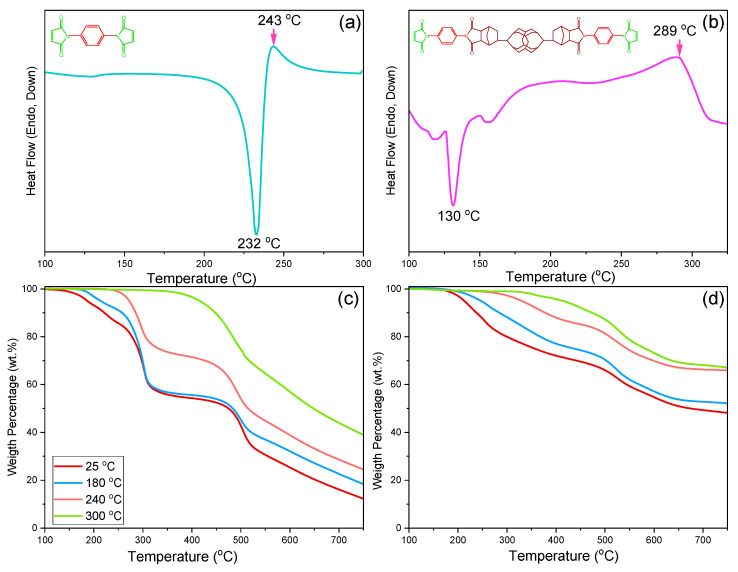
DSC analyses of (**a**) BMI and (**b**) DDSQ-BMI of the first heating run, TGA analyses of (**c**) BMI and (**d**) DDSQ-BMI after each thermal polymerization procedure.

DSC and TGA analyses are used to understand the thermal polymerization behavior of BMI and DDSQ-BMI monomers. Figure 2a,b display DSC thermograms of BMI and DDSQ-BMI after various thermal curing procedures. The obvious sharp endothermic peak at 232 °C corresponds to the melting temperature from pure BMI with high purity, and a weak broad exothermic peak at 243 °C is due to the addition polymerization of pure BMI. However, DDSQ-BMI shows a broad melting peak at 130 °C and other endothermal peaks at 156 and 117 °C, probably due to the two isomers during hydrosilylation from the DDSQ-ND monomer, as expected. The broad exothermic peak at 289 °C is also due to the addition polymerization of pure DDSQ-BMI. The higher exothermic peak of DDSQ-BMI than BMI is due to the DDSQ cage having a more rigid structure than the benzene ring is also as expected.

Figure 2c,d display the corresponding TGA analyses of BMI and DDSQ-BMI after various thermal curing procedures. Pure BMI or DDSQ-BMI displays three major thermal degradation procedures, where the first degradation procedure is due to the addition polymerization of double bonds at ca. 240 °C, the second degradation procedure comes from anhydride C=O units at ca. 300 °C, and the third degradation procedure at ca. 500 °C corresponds to BMI backbones, with temperature further increasing with crosslinking structures. Clearly, the *T*_d10_ value (10 wt% loss) and the char yield of the BMI and DDSQ-BMI monomers are 221 °C and 9.2 wt%, and 284 °C and 51.5 wt%, respectively. Both *T*_d_ and the char yield values increase upon the increase in thermal polymerization temperature for both BMI and DDSQ-BMI monomers because of the further addition polymerization. After thermal polymerization at 300 °C, the *T*_d_ value and the char yield of the BMI and DDSQ-BMI monomers are 447 °C and 35.1 wt%, and 478 °C and 68.0 wt%, respectively, confirming the DDSQ-BMI monomer features high thermal stability with significantly high char yield (68.0 wt%) due to the rigid DDSQ cage structure.

### 3.3. Thermal Curing Behavior of Epoxy/BMI and Epoxy/DDSQ-BMI Hybrids

**Figure 3:**
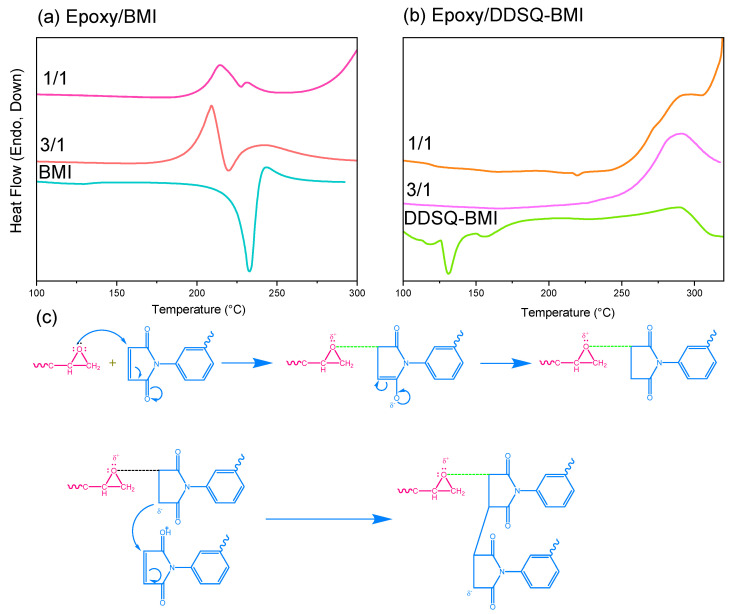
DSC analyses of (**a**) epoxy/BMI and (**b**) epoxy/DDSQ-BMI hybrids of first heating run, and (**c**) the thermal polymerization mechanism of epoxy with bismaleimide compound.

Figure 3 shows the DSC analyses of the epoxy/BMI and epoxy/DDSQ-BMI hybrids with various ratios at the heating rates of 20 °C/min. Pure BMI and DDSQ-BMI monomers have been discussed in Figure 2a,b; here, the epoxy/BMI and epoxy/DDSQ-BMI hybrids have the melting temperatures of BMI and DDSQ-BMI negated, indicating their complete miscibility with the epoxy resin. With the addition of epoxy resin into the BMI or DDSQ-BMI matrix, the thermal polymerization peaks are decreased to 209 and 214 °C for epoxy/BMI = 1/1 and 3/1, 281 and 290 °C for epoxy/DDSQ-BMI = 1/1 and 3/1, respectively. The reduction of thermal polymerization of the temperature peak is due to the formation of the zwitterion adduct between the oxirane ring of the epoxy resin and the double bond of BMI or DDSQ-BMI, as shown in Figure 3c [31], as well as the slight increase in the thermal polymerization temperature at a relative higher BMI or DDSQ-BMI, also confirmed by Musto et al. [30].

**Figure 4:**
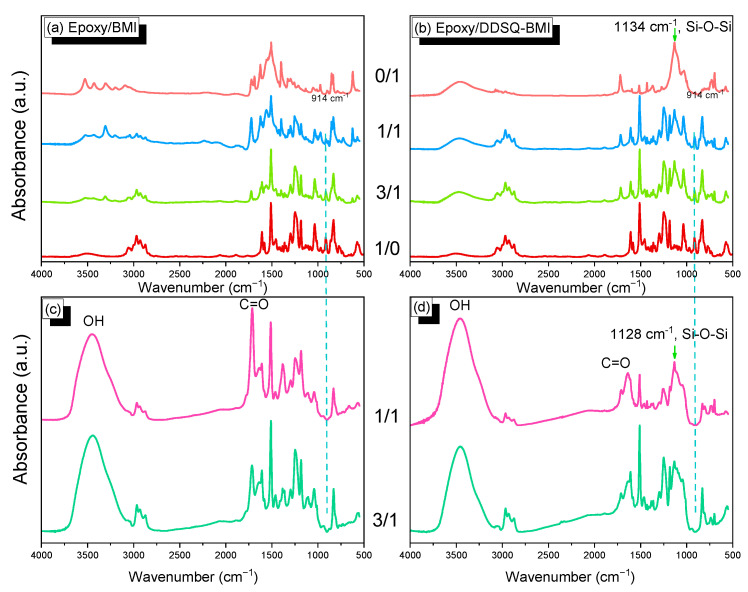
FTIR spectra of various (**a**,**c**) epoxy/BMI and (**b**,**d**) epoxy/DDSQ-BMI with 0/1, 1/1, 3/1, and 1/0 hybrids before (**a**,**b**) and after (**c**,**d**) thermal polymerization procedure.

To understand the thermal polymerization mechanism of these epoxy/BMI and epoxy/DDSQ-BMI hybrids, we use the FTIR analyses of these hybrids measured before and after thermal polymerization at 300 °C, as shown in Figure 4. Pure BMI shows a C=O imide absorption at 1688 and 1721 cm^−1^, and a C=C absorption at 1625 cm^−1^, as shown in (Figure 4a); pure DDSQ-BMI exhibits a C=O imide absorption at 1718 and 1773 cm^−1^ as mentioned previously, and a very weak C=C absorption at 1637 cm^−1^ (Figure 4b). Furthermore, the C=C-H absorption of pure BMI is located at 3093 cm^−1^ and the absorptions at 3304 and 3428 cm^−1^ are due to the overtone of C=O units of BMI, as shown in Figure 4a. Pure epoxy resin shows the epoxy absorption peak at 914 cm^−1^ due to the epoxy group in Figure 4a,b; various amounts of epoxy/BMI or epoxy/DDSQ-BMI hybrids in Figure 4a,b display the simple addition for both pure epoxy and pure BMI or pure DDSQ-BMI, indicating no chemical reaction takes place before thermal polymerization. From Figure 4c,d, the epoxy absorption at 914 cm^−1^ is completely gone and there is a very broad absorption at ca. 3450 cm^−1^ due to secondary OH stretching after thermal polymerization, indicating the ring-opening reaction of epoxy with the amine group from the BMI and DDSQ-BMI units [66]. Most importantly, these secondary OH groups could form the intermolecular hydrogen bonding interaction with the Si-O-Si groups of DDSQ from 1134 cm^−1^ for pure DDSQ-BMI, which was shifted to 1128 cm^−1^, as blending with epoxy resin after thermal polymerization enhances the miscibility, thermal, and mechanical properties of polymer matrix, as widely discussed in our previous works [67,68].

**Figure 5:**
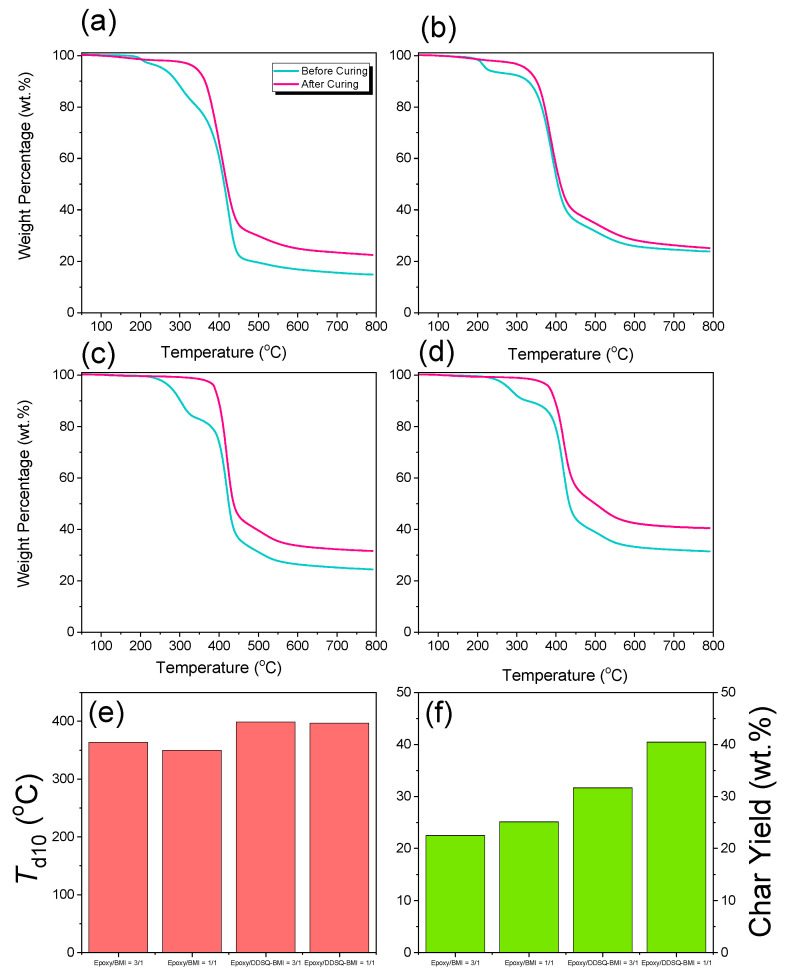
TGA analyses of (**a**) epoxy/BMI = 3/1, (**b**) epoxy/BMI = 1/1, (**c**) epoxy/DDSQ-BMI = 3/1, and (**d**) epoxy/DDSQ-BMI = 1/1 hybrids before and after the thermal polymerization procedure, and the corresponding *T*_d_ value (**e**) and char yield (**f**).

TGA analyses of various amounts of epoxy/BMI or epoxy/DDSQ-BMI hybrids before and after the thermal polymerization procedure at 300 °C is shown in Figure 5a–d. Clearly, both *T*_d_ and char yield values are increased after the thermal polymerization procedure, indicating that the crosslinking structure is formed into the epoxy resin, which could enhance the thermal properties in these cases. Both the *T*_d_ and char yield values are also summarized in Figure 5e–f. Firstly, the increase in BMI and DDSQ-BMI into epoxy resin does not increase the *T*_d_ value; however, it does increase the char yield compared to epoxy/BMI or epoxy/DDSQ-BMI hybrids = 3/1 and 1/1 ratios. Secondly, incorporation of DDSQ into BMI could both enhance the *T*_d_ value and char yield in both epoxy/BMI and epoxy/DDSQ-BMI hybrids = 3/1 and 1/1 ratios because of the rigid inorganic DDSQ cage structure. The DDSQ cage structure can reduce the organic material decomposition due to the covalent bond of DDSQ into BMI units; this restricts the thermal motion of epoxy and BMI units through the formation of a network structure with this inorganic DDSQ cage. The DDSQ may possess the ceramic inorganic layer during the combustion at the early stage because of its low surface free energy property [67], and this DDSQ layer could protect and limit the heat transfer from the O_2_ diffusion in epoxy or BMI resin. Compared with epoxy/BMI and epoxy/DDSQ-BMI = 1/1 system, the *T*_d_ value is increased from 350 to 397 °C and the char yield is significantly increased from 25.1 to 40.4 wt% after the incorporation DDSQ units into the BMI monomer. Furthermore, we also compared this with epoxy/BMI or epoxy/DDSQ-BMI hybrids after thermal polymerization at 300 °C and sitting at 250 °C by TGA analyses, as shown in Figure S2. Clearly, the DDSQ cage into the BMI could enhance the thermal stability after 24 h where the char yield of epoxy/DDSQ-BMI = 3/1 is 93.6 wt%; however, the epoxy/BMI = 3/1 is only 85.8 wt%.

**Figure 6:**
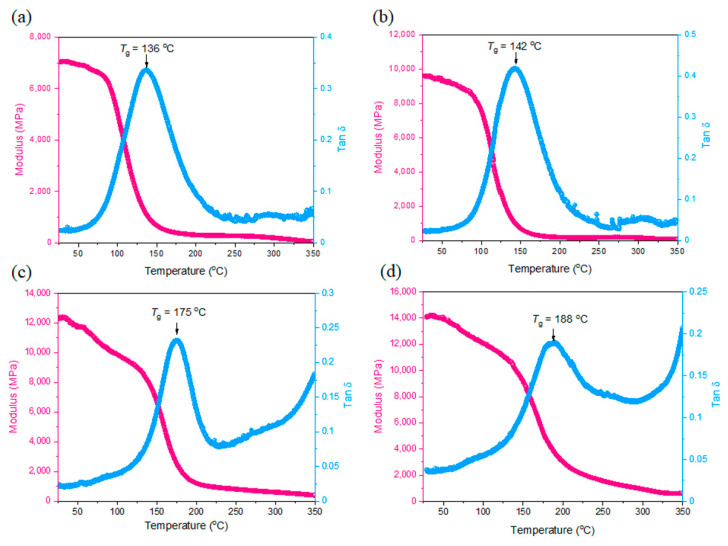
DMA analyses of (**a**) epoxy/BMI = 3/1, (**b**) epoxy/BMI = 1/1, (**c**) epoxy/DDSQ-BMI = 3/1, and (**d**) epoxy/DDSQ-BMI = 1/1 hybrids after thermal polymerization procedure where pink line is storage modulus and blue line is tan δ.

Figure 6 displays the DMA thermal analyses of epoxy/BMI and epoxy/DDSQ-BMI hybrids with various ratios regarding the storage modulus (*E*′) and loss tan δ results after thermal polymerization at 300 °C. The initial storage of epoxy/BMI = 3/1 was 7074 MPa at 25 °C and the loss tan δ peak, relative to the glass transition temperature, was 136 °C, as shown in Figure 6a. Increasing the BMI concentration into epoxy resin as epoxy/BMI = 1/1 mixture, the initial *E*′ value and tan δ peak were both increased to 9597 MPa and 142 °C, respectively, as shown in Figure 6b. After the incorporation of DDSQ into BMI, the initial *E*′ value and tan δ peak were both increased to 12,416 MPa and 175 °C (Figure 6c) and 14,044 MPa and 188 °C (Figure 6d) for epoxy/DDSQ-BMI = 3/1 and 1/1, respectively. Both *E*′ value and tan δ peak were increased because the cubic DDSQ cage is rigid, and the *T*_g_ value also correspond to the cross-linking density and mobility of epoxy resin based on the nano-reinforcement effect [46,47].

**Figure 7:**
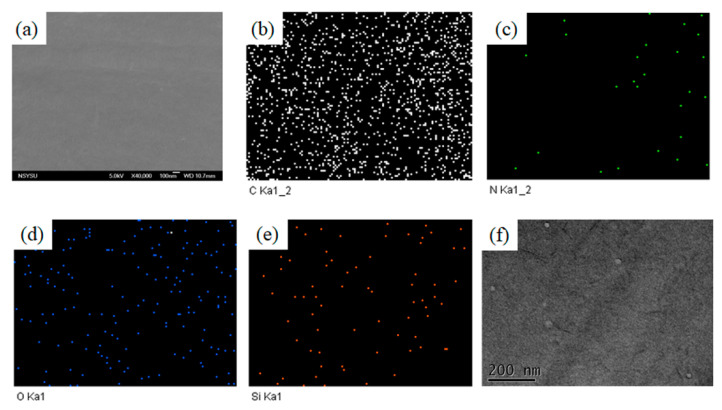
(**a**) SEM, (**b**) C, (**c**) N, (**d**) O, and (**e**) Si-mapping and (**f**) TEM image of epoxy/DDSQ-BMI = 3/1 after thermal polymerization.

Finally, the DDSQ-BMI dispersion into the epoxy resin was investigated by SEM analysis. Figure 7a presents SEM imagery of the epoxy/DDSQ-BMI = 3/1 mixture after thermal polymerization; the featureless morphology without phase separation indicates that DDSQ is dispersed homogeneously into the epoxy resin. In addition, the C, N, O, and Si-mapping (Figure 7b–e) also indicate uniform dispersion of the DDSQ nanoparticles on the epoxy surfaces, where the red points are the DDSQ-rich domains, which is also confirmed by TEM image in Figure 7f. This uniform dispersion of inorganic DDSQ nanoparticles in the epoxy resin could decrease the chain mobility and enhance the thermal degradation, which is consistent with DMA and TGA analyses.

## 4. Conclusions

We have successfully prepared a DDSQ-BMI monomer by using many chemical reactions featuring high thermal stabilities such as *T*_d10_ value (478 °C) and char yield (68 wt%) after a thermal polymerization procedure due to the rigid inorganic DDSQ cage created by addition polymerization. Furthermore, the inorganic DDSQ-BMI cage could also be dispersed homogeneously in epoxy resin based on SEM and TEM image analyses, which could decrease the chain mobility and thus the storage modulus; the *T*_g_ and *T*_d_ values of these hybrids could be significantly improved after incorporation of DDSQ-BMI into the epoxy resin due to the hydrogen bonding interaction between the DDSQ and OH group of epoxy after thermal polymerization based on FTIR analyses. Based on TGA and DMA analyses, the *T_g_* and *T*_d_ values could be enhanced to 188 °C and 397 °C, respectively, due to the physical rigid inorganic DDSQ cage structure, which was much higher than that of the typical DGEBA type epoxy resin with various organic curing agents.

## Data Availability

Not applicable.

## Supplementary Materials

The following supporting information can be downloaded at: https://www.mdpi.com/article/10.3390/polym14122380/s1. Figure S1: 1H NMR of BMI. Figure S2. TGA analyses of Epoxy/BMI and Epoxy/DDSQ-BMI.
