# Supplementary material for: Double-Decker-Shaped Polyhedral Silsesquioxanes Reinforced Epoxy/Bismaleimide Hybrids Featuring High Thermal Stability
Source: Polymers (Basel). 2022 Jun 12;14(12):2380. doi: 10.3390/polym14122380 (PMC9229952; doi:10.3390/polym14122380)
Supplement: Supplementary file 1 [file polymers-14-02380-s001.zip › polymers-1758075-supplementary.pdf]

Supporting Information

Double-Decker–Shaped Polyhedral Silsesquioxanes Reinforced Epoxy/Bismaleimide Hybrids Featuring High Thermal Stability

Wei-Cheng Chen,[1] Zih-Yu Chen,[1] Yuxia Ba,[2] Bingyang Wang,[2] Guofei Chen,[3] Xingzhong Fang,[3,*] and Shiao-Wei Kuo[1,4,*]

1 Department of Materials and Optoelectronic Science, Center of Crystal Research and Center for Functional Polymers and Supramolecular Materials, National Sun Yat-Sen University, Kaohsiung 80424, Taiwan; chwei556@gmail.com (C.W.C.); b073100051@student.nsysu.edu.tw (Z.Y.C.); kuosw@faculty.nsysu.edu.tw (S.W.K.)

2 Dongying Xinbang Electronic Technology Co., Ltd., Dongying, Shandong 257000, China; 472200567@qq.com (Y.B.); wang8957wang@126.com (B.W.)

3 Ningbo Institute of Materials Technology and Engineering, Chinese Academy of Sciences, Ningbo, Zhejiang 315201, China; gfchen@nimte.ac.cn (G.C.); fxzhang@nimte.ac.cn (X.F.)

4 Department of Medicinal and Applied Chemistry, Kaohsiung Medical University, Kaohsiung 807, Taiwan; kuosw@faculty.nsysu.edu.tw (S.-W.K.)

*****Correspondence: fxzhang@nimte.ac.cn (X.F.) and kuosw@faculty.nsysu.edu.tw (S.W. K.)

Characterization

^1H spectra were recorded on a INOVA 500 MHz NMR spectrometer, with $CDCl_3$-*d* as an external standard. The IR spectra were measured with a Bruker Tensor 27 FTIR spectrophotometer using the conventional crystal KBr disk method. 32 scans were collected at a spectral resolution of 4 cm^{-1}. Dynamic mechanical behavior of cured sample was studied using a Du-Pont 2980 dynamic mechanical analyzer. Cured sample was polished to ≈ 3.0 × 13.0 × 30.0 mm and mounted on a single cantilever clamp. The mechanical properties were measured under nitrogen in step mode every 5 ℃ from 25 to 350 ℃ at frequency of 1 Hz. The thermal stability of the samples was characterized by using a TA Q-50 Thermogravimetric Analyzer operating under a nitrogen atmosphere. The cured sample ca. 7 mg was placed in a Pt cell and heated at a rate of 20 ℃ /min from 30 to 800 ℃ at a nitrogen flow rate of 60 mL/min. The dynamic curing kinetics was studied using a TA Q-20 instrument Differential Scanning Calorimeter operating under a nitrogen atmosphere. The sample (ca. 7 mg) was placed in a sealed aluminum sample pan. Dynamic curing scan were conducted from 30 ℃ to 350 ℃ at a heating rate of 20 ℃ /min.

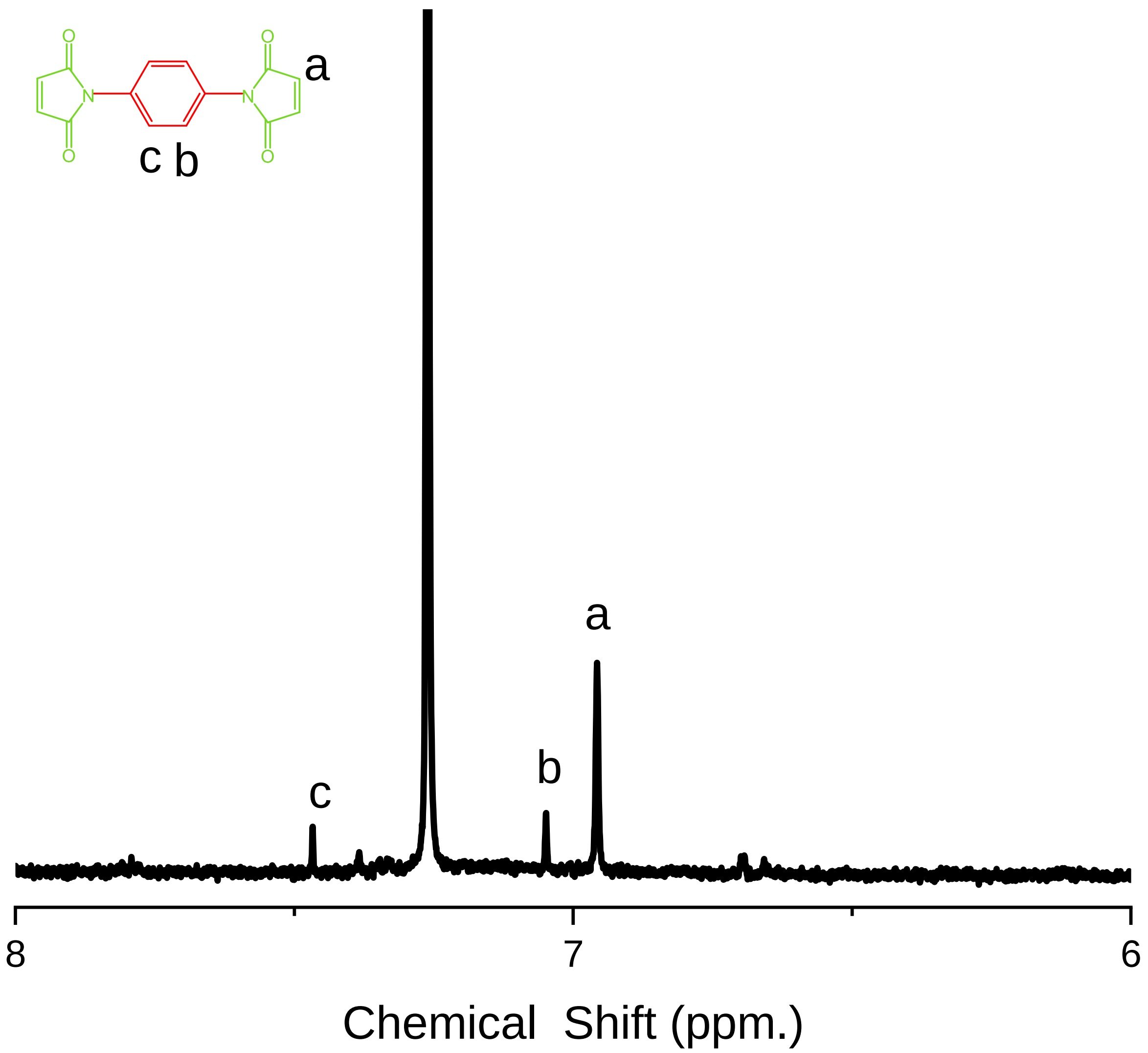

Figure S1: ^{1}H NMR spectrum of BMI

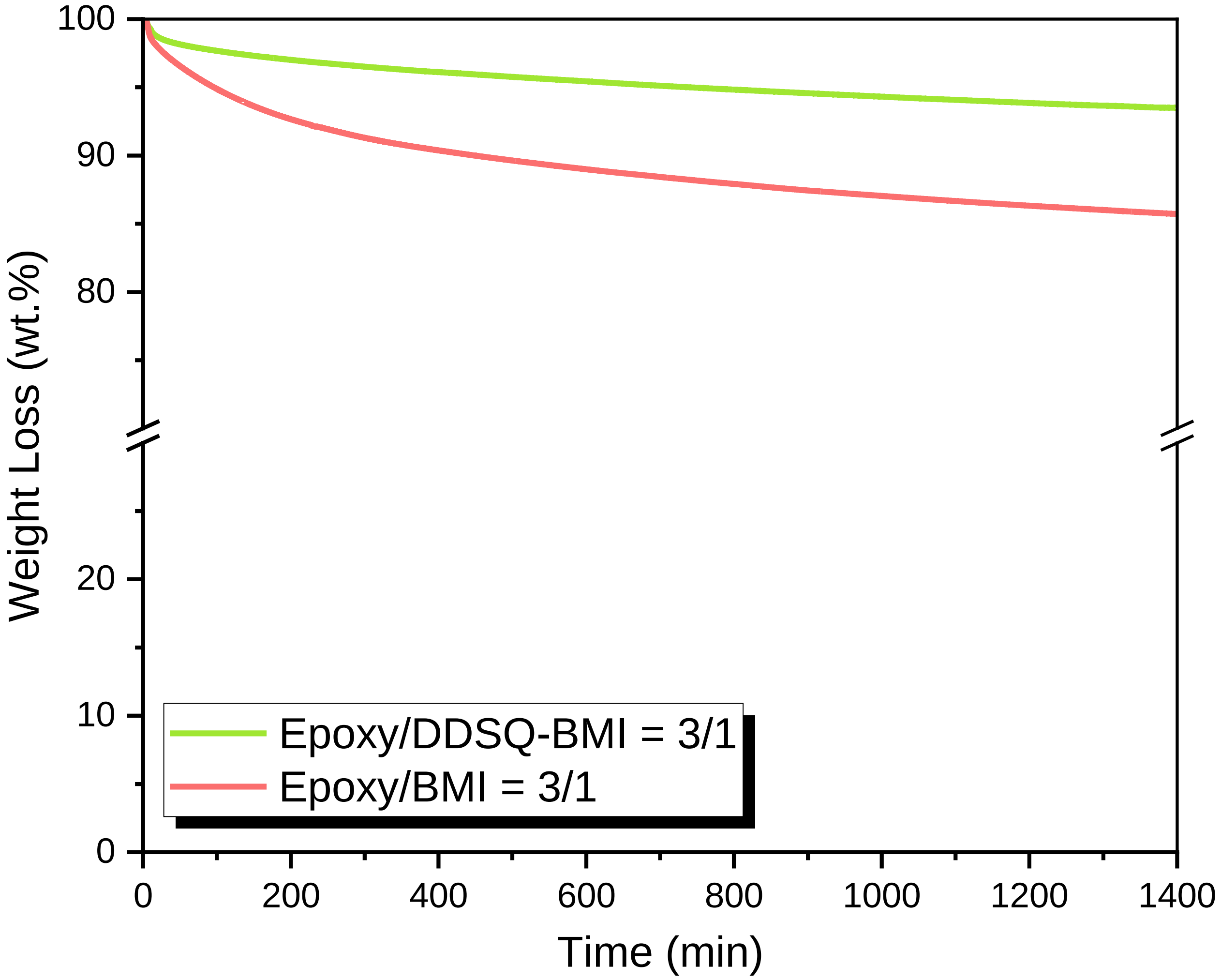

Figure S2: TGA analyses of Epoxy/BMI and Epoxy/DDSQ-BMI = 3/1 after thermal polymerization and the remained at 250 °C for 24 hr
